# Investigation on FAB Morphology Evolution and Pd Redistribution Behavior in Palladium-Coated Copper Wires During Electronic Flame-Off (EFO) Process

**DOI:** 10.3390/mi17091058

**Published:** 2026-09-04

**Authors:** Junling Fan, Haoyang Wang, Yongzhen Sun, Jun Cao, Weilong Liu

**Affiliations:** 1 School of Chemical and Environmental Engineering, Jiaozuo University, Jiaozuo 454000, China; 2 Henan Jinda Wire Co., Ltd., Luoyang 471922, China; 3 School of Mechanical and Power Engineering, Henan Polytechnic University, Jiaozuo 454003, China

**Keywords:** palladium-plated copper wire, free air ball, element redistribution, microelectronic packaging

## Abstract

Compared with bare copper wire, palladium-coated copper (PCC) wire is widely used in microelectronic packaging due to its improved oxidation resistance and enhanced reliability. However, the formation mechanism of free air balls (FABs) and the redistribution behavior of Pd during the electronic flame-off (EFO) process, particularly under different Pd coating thicknesses and processing conditions, have not yet been fully understood. In this work, four types of 1 mil PCC wires with Pd coating thicknesses of 60, 80, 100, and 120 nm were systematically investigated to study the influence of EFO parameters on FAB morphology and Pd redistribution behavior. SEM, FIB, and EDS analyses were employed to provide experimental insights into the coupled relationship between transient thermal input, internal pore distribution and elemental segregation evolution, and Pd redistribution behavior. The results show that the preferred FAB morphology is obtained at 54 mA and 580 μs, with a diameter-to-wire ratio of approximately 2. With increasing Pd coating thickness, the exposed copper area on the FAB surface decreases from 13% to 6%, while the Pd-deficient region gradually shifts toward the bottom of the FAB. This study provides experimental insights into the Pd redistribution behavior during FAB formation under different EFO conditions, which may contribute to the optimization of Pd-coated Cu bonding wires.

## 1. Introduction

Microelectronic packaging serves as a key interconnection technology between chips and external circuits, playing a critical role not only in mechanical protection but also in determining the electrical, thermal, and reliability performance of devices. With the continuous trend toward high-density integration, miniaturization, and enhanced reliability, increasingly stringent requirements have been imposed on bonding wire materials and their ball formation processes [1,2,3,4].

Gold wire remains the most widely used bonding wire material due to its excellent oxidation resistance, corrosion resistance, and stable interfacial properties. However, the continuously rising cost of gold has significantly limited its large-scale application. In contrast, copper wire has emerged as a promising alternative owing to its superior electrical and thermal conductivity as well as its low cost. Nevertheless, bare copper wire is prone to oxidation during the electronic flame-off (EFO) process and is also susceptible to interfacial corrosion and failure under service conditions [5,6,7].

To address these issues, palladium-coated copper (PCC) wire has been proposed as an effective solution [8]. The Pd coating significantly improves oxidation and corrosion resistance and suppresses the excessive growth of intermetallic compounds (IMCs) at the Cu/Al interface, thereby enhancing long-term reliability. In addition, Pd has been reported to improve interfacial stability and reduce environmental degradation effects [9,10,11].

During the EFO process, the wire tip is melted to form a free air ball (FAB), whose geometry and internal structure strongly influence bonding reliability. Previous studies have shown that significant temperature gradients and melt convection exist during FAB formation and that Pd redistributes under the combined effects of surface tension and gravity. Moreover, the Pd-deficient region generated by the second bond breakage can be inherited to the FAB surface, resulting in non-uniform Pd coverage [12,13,14,15].

Further investigations indicate that EFO parameters, such as current and time, not only determine the overall FAB morphology but also induce asymmetric melt flow within the molten pool, leading to complex Pd migration both on the surface and inside the FAB. Under high-current conditions, enhanced convection may even develop into convective flow channels, driving Pd enrichment near the neck region or localized areas, while copper-exposed regions tend to form elsewhere. However, the three-dimensional migration pathways of Pd within the FAB and their coupling with void structures remain insufficiently understood, particularly under different Pd coating thickness conditions [16,17,18,19,20].

Based on this, four types of 1 mil PCC wires with Pd coating thicknesses of 60, 80, 100, and 120 nm were selected. The preferred EFO parameters for each material were determined using a FAB diameter-to-wire diameter ratio close to 2 as the evaluation criterion [21,22,23,24]. On this basis, the influence of EFO current on FAB morphology and Pd redistribution behavior on both the surface and interior was systematically investigated under fixed discharge time conditions. Furthermore, cross-sectional analysis was conducted to elucidate the coupling relationship between Pd redistribution and internal void structures [25,26,27,28,29,30].

Previous studies mainly focused on the influence of Pd coating thickness and EFO parameters on FAB morphology and surface Pd coverage. However, the internal cross-sectional pore characteristics and its coupling with elemental segregation inside the transient molten pool have not been systematically characterized by cross-sectional analysis. Distinct from our previous research [1] which primarily focused on macroscopic morphology, this work provides quantitative experimental insights into the internal structural evolution and internal Pd redistribution behavior via FIB-SEM and EDS.

## 2. Tests and Methods

**Figure 1:**
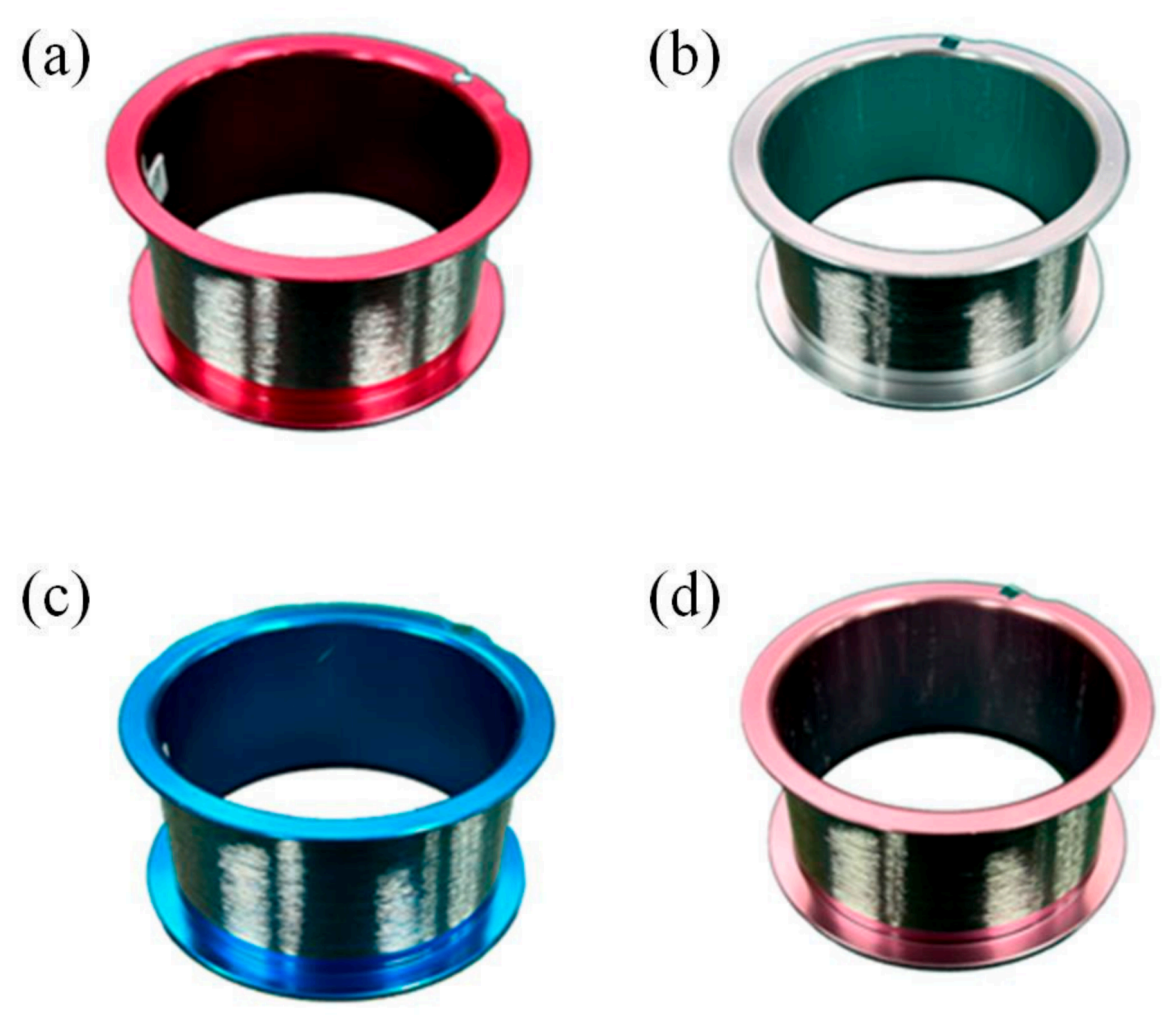
PCC wires with different thicknesses: (**a**) bonding wire A; (**b**) bonding wire B; (**c**) bonding wire C; (**d**) bonding wire D.

In this study, four types of palladium-coated copper (PCC) bonding wires with different Pd coating thicknesses (60, 80, 100, and 120 nm) were selected and designated as wires A, B, C, and D, respectively, as shown in Figure 1. The nominal Pd coating thicknesses were provided by the wire manufacturer based on coating process calibration. The present study focuses on comparative behavior among wires with different commercial Pd thickness levels. The ball formation experiments were conducted using an FB-988 fully automatic wire bonder (KAIJO Co., Tokyo, Japan).

To investigate the influence of EFO parameters on FAB morphology and Pd distribution in PCC wires with different Pd coating thicknesses, electronic flame-off (EFO) balling experiments were carried out. The discharge current was varied from 44 to 64 mA with a step of 5 mA, while the discharge time ranged from 500 to 660 μs with a step of 40 μs. Each parameter set was independently repeated 40 times to ensure statistical reliability and experimental reproducibility. Representative images and quantitative data in the figures were selected from these repeat experiments. The quantitative morphology, exposed Cu area, pore distribution, and EDS results presented in this study are based on measurements from at least 3 independently formed FABs, with the mean and standard deviation calculated for the corresponding data. All parameter combinations were applied to the four PCC wire types with different Pd thicknesses (60, 80, 100, and 120 nm), as summarized in Table 1. The preferred conditions of this study are within the parameters of the experiment.

After ball formation, the surface morphology of the FABs was characterized using a Merlin Compact scanning electron microscope (SEM, Carl Zeiss NTS GmbH, Oberkochen, Germany) to evaluate the influence of EFO parameters on FAB evolution. To further analyze Pd distribution on the FAB surface, selected samples prepared under preferred EFO conditions were immersed in 3% FeCl_3_ solution for 10 s. The resulting corrosion patterns were used to assess the uniformity of Pd coverage. Under fixed preferred discharge time conditions, FABs were also prepared at different discharge currents, and cross-sectional microstructures were examined using a Helios 5 CX focused ion beam–scanning electron microscope (FIB-SEM) to investigate Pd redistribution behavior within the FAB. During FAB formation, nitrogen was used as a protective atmosphere to minimize copper oxidation at elevated temperatures. To avoid the influence of the second bond on FAB morphology, the bonding parameters were fixed as follows: ultrasonic power 85 mW, bonding time 38 ms, and bonding force 40 gf. This step was only used to fix the wire position on the pad and did not contribute to electrical interconnection.

## 3. Results and Discussion

Section 3.1 and Section 3.2 present the macroscopic morphology and surface Pd coverage trends in FABs under different EFO conditions. While these trends are consistent with those in previous studies (such as Ref. [1]), they serve as a necessary basis for the subsequent analysis. The primary novelty of the present work lies in Section 3.3, where the internal cross-sectional pore structures and the internal Pd/Cu redistribution are systematically correlated for the first time via FIB-SEM and EDS.

### 3.1. Optimization of EFO Parameters and Evolution of FAB Morphology

Prior to first bond formation, the wire tip is melted by an electronic flame-off (EFO) process to form a free air ball (FAB). The FAB morphology plays a critical role in determining bonding interface strength and, consequently, the electrical and mechanical reliability of the device. Therefore, the optimization of EFO parameters is necessary to obtain FABs with uniform morphology, high sphericity, and good symmetry. It has been reported that reliable first bonds are typically achieved when the FAB diameter is 1.5–2.5 times the wire diameter. In this study, a diameter-to-wire ratio close to 2 was adopted as the evaluation criterion for preferred FAB morphology [31,32].

**Figure 2:**
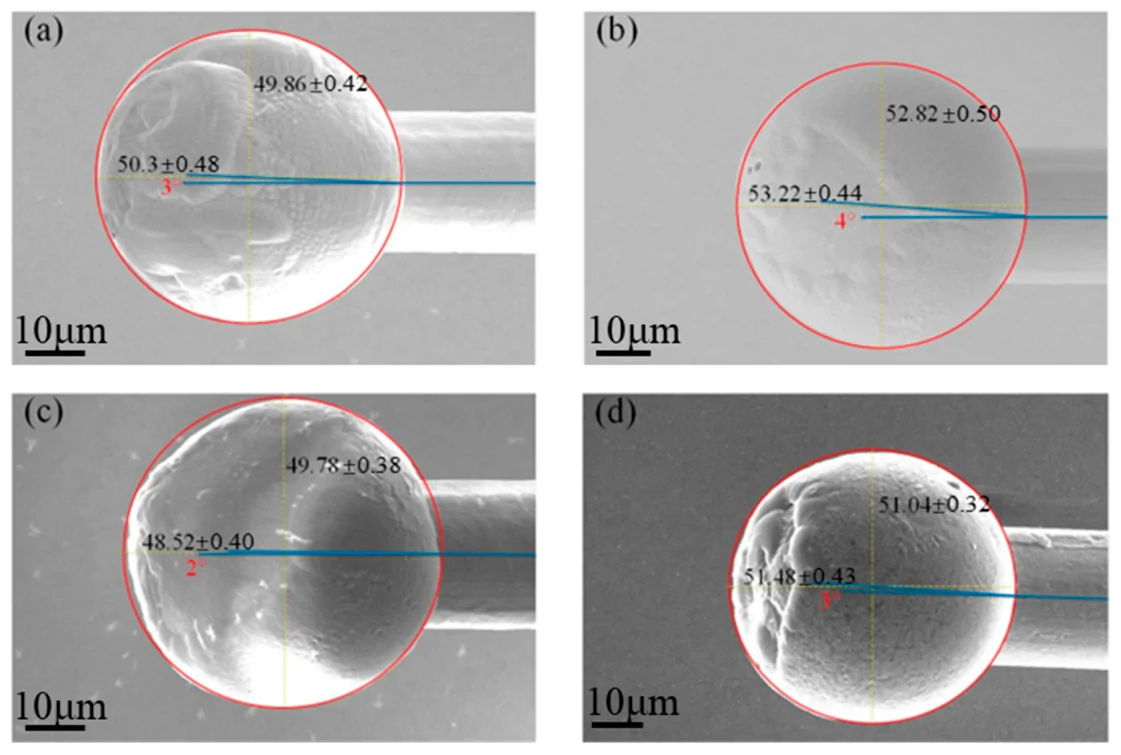
Measurement of free air ball (FAB) diameter and ball asymmetry angle for four types of bonding wires formed under burning ball time of 580 μs and burning ball current of 54 mA: (**a**) bonding wire A; (**b**) bonding wire B; (**c**) bonding wire C; (**d**) bonding wire D.

As shown in Figure 2, under the conditions of 580 μs discharge time and 54 mA discharge current, the FABs formed from the four PCC wires exhibit diameter-to-wire ratios close to 2. The measured FAB diameters and deviation angles are presented in Figure 2, and the corresponding symmetry and roundness parameters were further calculated, as summarized in Table 2. Under identical EFO conditions (54 mA, 580 μs), the FAB geometrical parameters of all four wires approach the preferred range. As listed in Table 2, the FAB diameters, symmetry, roundness, and deviation angles show good consistency, with wire D exhibiting the most balanced overall morphology.

$D_{R}$ and roundness $D_{RD}$ are defined as follows:

(1) $$D_{R}\;=\;D_{A}/D_{B}$$

(2) $$D_{RD}=|D_{A}-D_{B}|/\left(\frac{\left(D_{A}+D_{B}\right)}{2}\right)$$

where $D_{A}$ is the FAB diameter measured perpendicular to the wire axis, and $D_{B}$ is the diameter measured along the wire axis.

**Figure 3:**
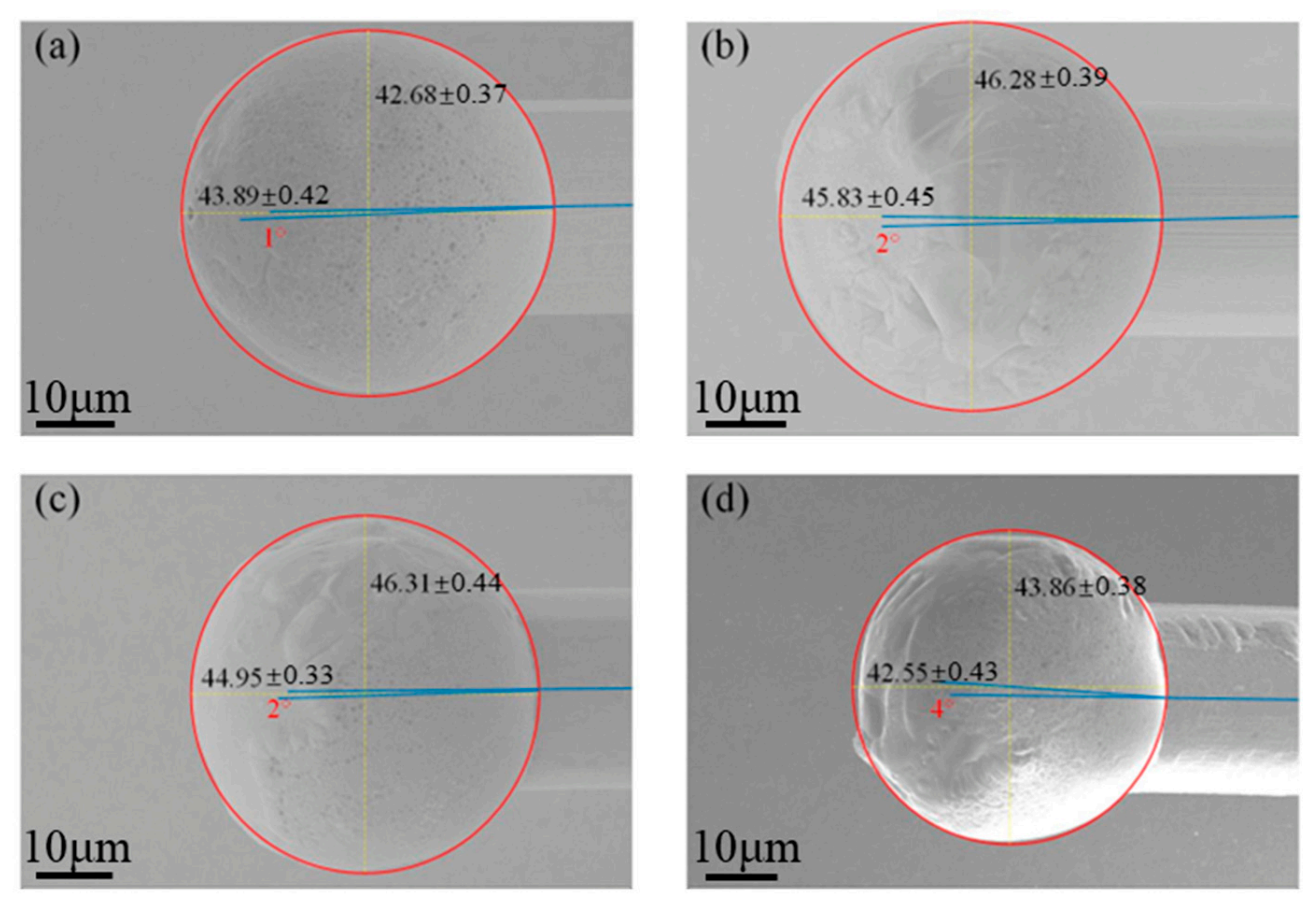
Measurement of free air ball (FAB) diameter and ball asymmetry angle for four types of bonding wires formed under burning ball time of 500 μs and burning ball current of 54 mA: (**a**) bonding wire A; (**b**) bonding wire B; (**c**) bonding wire C; (**d**) bonding wire D.

However, as shown in Figure 3, when the discharge time is reduced to 500 μs, all FABs exhibit varying degrees of deviation, surface depression, and groove-like defects. These defects are attributed to insufficient energy input during EFO, which prevents the complete melting of the wire tip and results in unstable solidification under surface tension and gravity [33,34,35].

**Figure 4:**
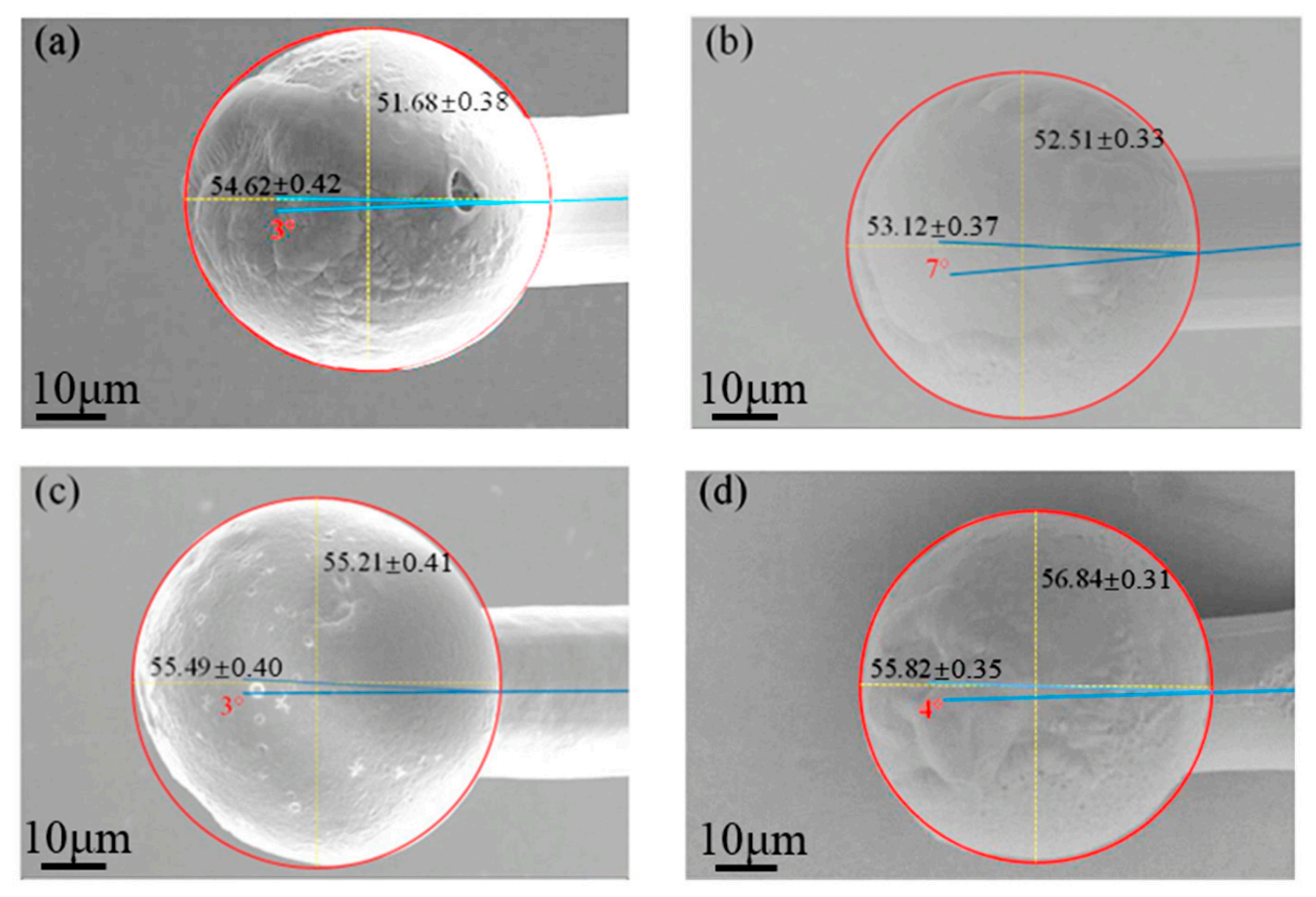
Measurement of free air ball (FAB) diameter and ball asymmetry angle for four types of bonding wires formed under burning ball time of 660 μs and burning ball current of 54 mA: (**a**) bonding wire A; (**b**) bonding wire B; (**c**) bonding wire C; (**d**) bonding wire D.

When the discharge time is increased to 660 μs (Figure 4), more severe morphological instabilities are observed. Wire A shows pronounced surface grooves and a large void in the upper-middle region of the FAB. Wire B exhibits layered structures in the lower region. Wire C develops a sharp ball morphology, while wire D shows distinct grooves at the bottom of the FAB. These results indicate that excessive discharge duration leads to over-melting and excessive melt fluidity, thereby destabilizing FAB geometry and reducing symmetry and roundness.

**Figure 5:**
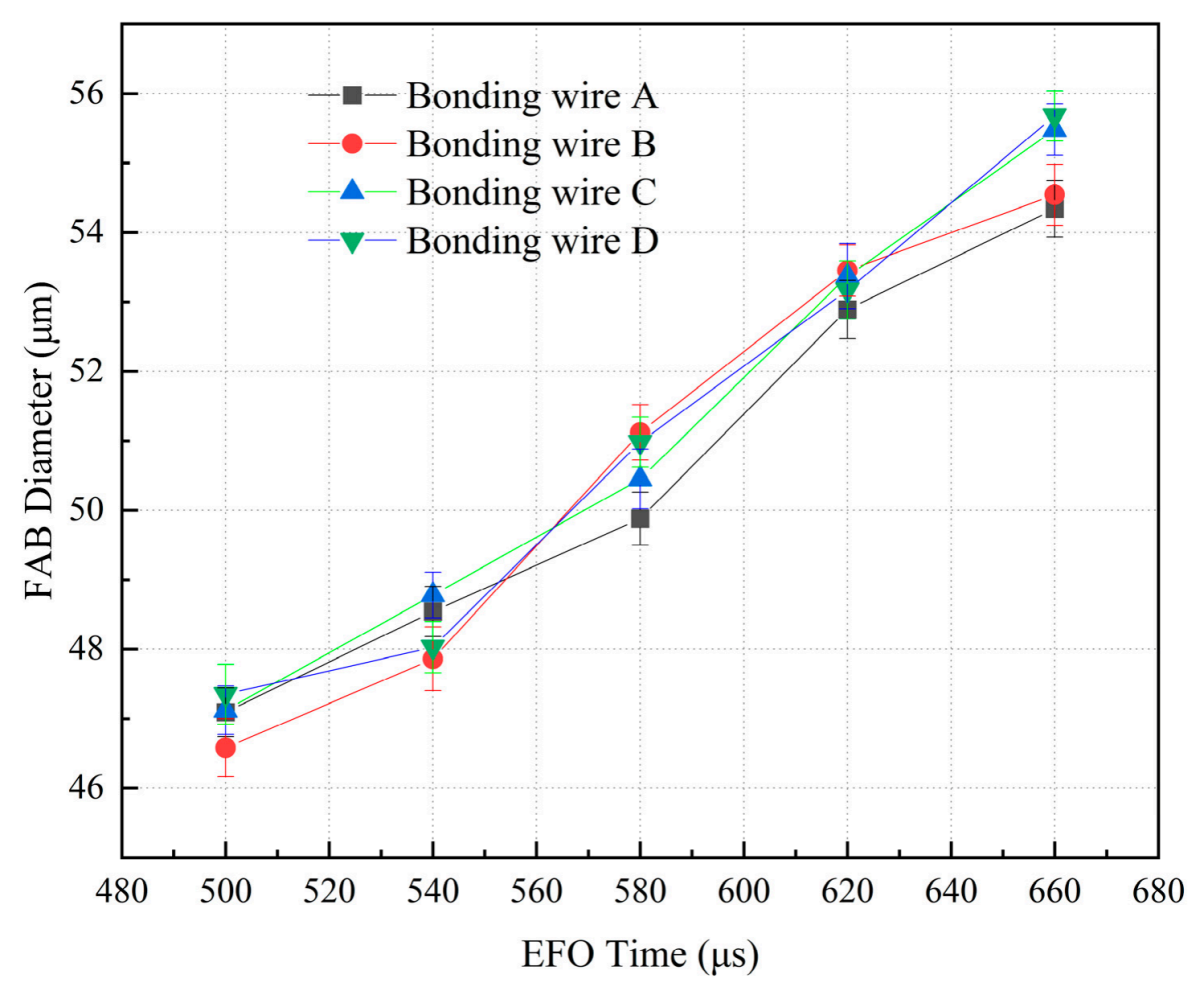
Evolution of free air ball (FAB) diameter as function of burning ball time for four types of bonding wires under burning ball current of 54 mA.

For each parameter, the FAB diameter is taken as the average of the values. Figure 5 shows that the FAB diameter increases monotonically with increasing discharge time from 500 to 660 μs for all wire types. Among these conditions, 580 μs and 54 mA are identified as the preferred parameter combination for further analysis.

**Figure 6:**
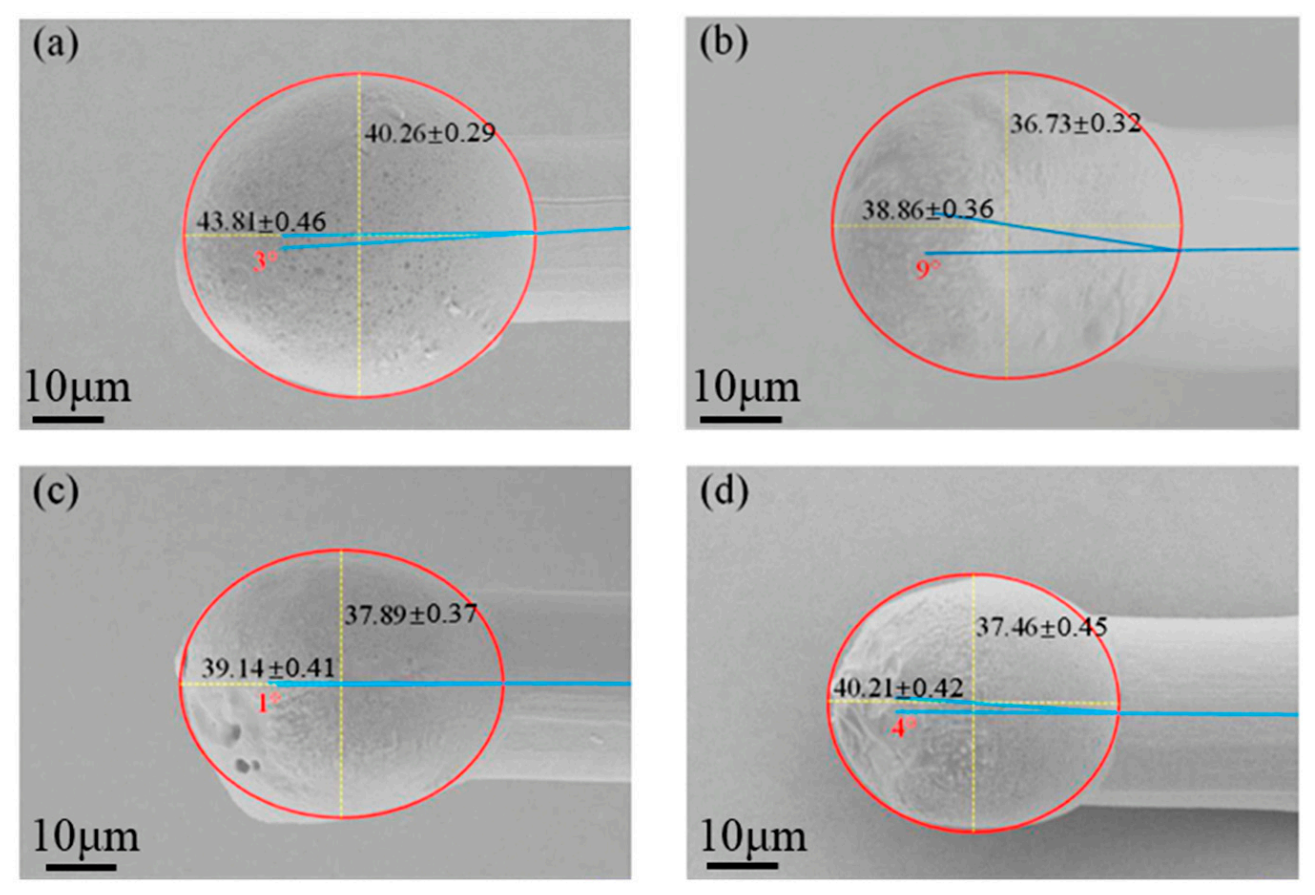
Measurement of free air ball (FAB) diameter and ball asymmetry angle for four types of bonding wires formed under burning ball time of 580 μs and burning ball current of 44 mA: (**a**) bonding wire A; (**b**) bonding wire B; (**c**) bonding wire C; (**d**) bonding wire D.

During the EFO process, the wire tip is rapidly heated by transient arc discharge and subsequently melts to form the FAB. The melting behavior is mainly determined by the electrical energy input, discharge duration, and heat dissipation conditions. Increasing the discharge time or current generally enhances the energy supplied to the wire tip, resulting in a larger molten volume and promoting FAB growth. However, excessive energy input may induce unstable molten flow and deteriorate FAB morphology. At a fixed discharge time of 580 μs and a low current of 44 mA (Figure 6), the FABs exhibit elliptical morphologies. Wire A shows a sharp ball defect, wire B is insufficiently melted with poor roundness, and wires C and D display surface depressions at the FAB bottom. These observations indicate that insufficient current results in inadequate melting and unstable FAB formation.

**Figure 7:**
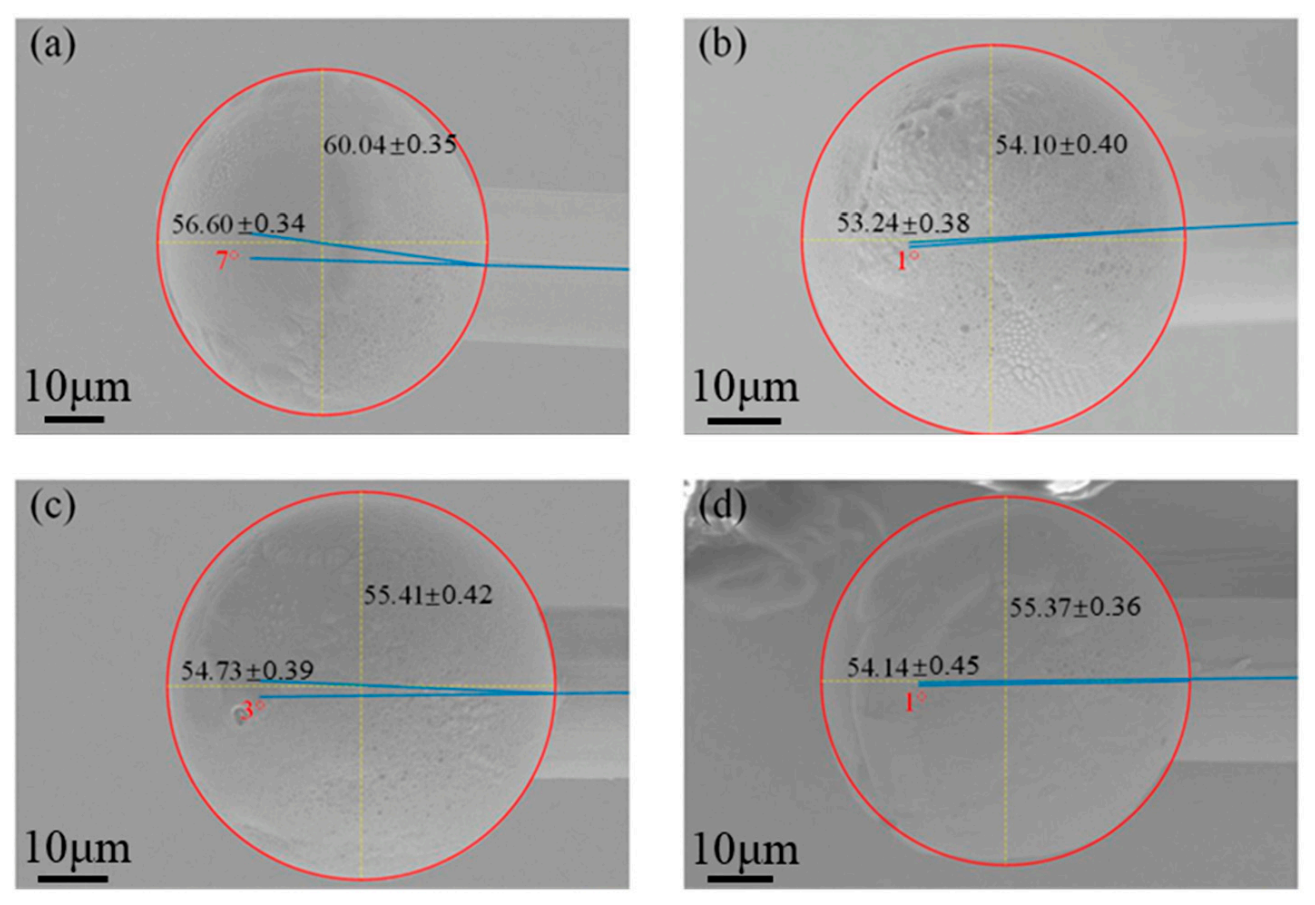
Measurement of free air ball (FAB) diameter and ball asymmetry angle for four types of bonding wires formed under burning ball time of 580 μs and burning ball current of 64 mA: (**a**) bonding wire A; (**b**) bonding wire B; (**c**) bonding wire C; (**d**) bonding wire D.

When the current is increased to 64 mA (Figure 7), FAB morphology is generally improved, with enhanced smoothness and better symmetry. However, localized defects remain, including surface depressions in wires A and C, large voids in wire B, and layered structures in wire D. This suggests that excessive thermal input enhances melting but also induces unstable melt flow and structural defects.

**Figure 8:**
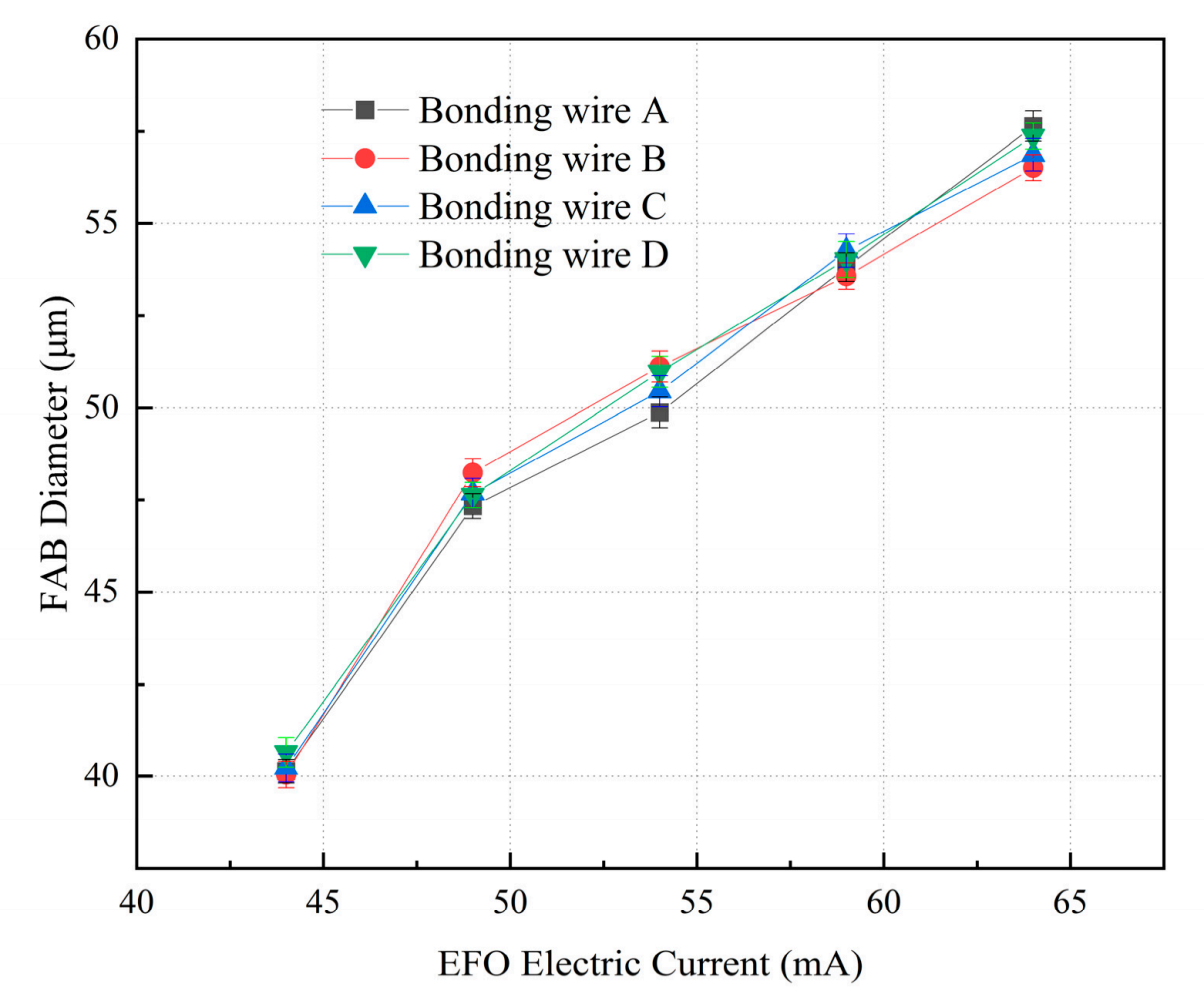
Variation in free air ball (FAB) diameter with burning ball current for four types of bonding wires under burning ball time of 580 μs.

As shown in Figure 8, the FAB diameter strongly depends on discharge current at a fixed time of 580 μs. Taking wire D as an example, the FAB diameter at 44 mA is 40.65 μm, which is 20.26% lower than the preferred condition. When the current increases to 49 mA, the reduction decreases to 6.57%, indicating a rapid increase in FAB size in the low-current regime. This confirms that increasing current significantly enhances melting and promotes FAB formation.

Overall, the evolution of FAB morphology is governed by a balance between thermal input, melting extent, and melt flow stability. Insufficient energy leads to under-melted and defective FABs, whereas excessive energy induces over-fluidity and structural instability. A preferred EFO window is identified at 54 mA and 580 μs, where all wire types exhibit superior sphericity, symmetry, and minimal deviation angle.

### 3.2. Evolution of Pd Distribution on FAB Surface and Influence of Burning Ball Current

**Figure 9:**
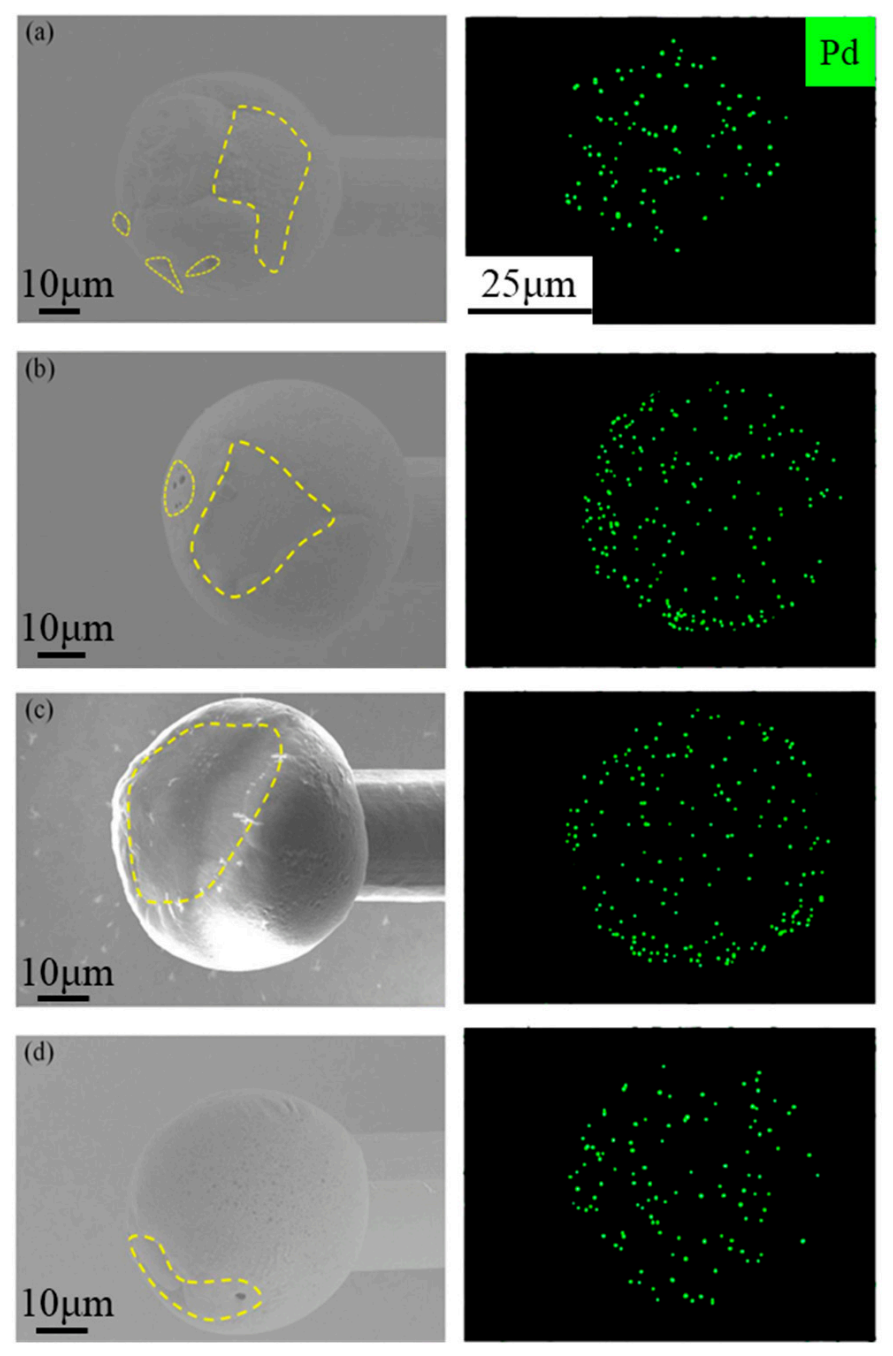
Scanning electron microscopy images of free air balls formed from four wire types after corrosion treatment under a burning current of 54 mA and a burning time of 580 μs, together with the corresponding Pd element distribution on the free air ball surface: (**a**) bonding wire A; (**b**) bonding wire B; (**c**) bonding wire C; (**d**) bonding wire D.

Under the preferred burning ball condition (burning ball current 54 mA, burning ball time 580 μs), the surface morphologies of FABs formed from four Pd-coated copper wires with different Pd layer thicknesses after corrosion are as shown in Figure 9. The yellow dashed regions correspond to Cu-exposed areas caused by insufficient Pd coverage. The results indicate that for wire A, Cu-exposed regions are mainly located in the central and neck regions of the FAB; for wire B, they are concentrated in the lower-middle region; and for wires C and D, these regions progressively shift toward the bottom of the FAB and exhibit improved continuity. With increasing Pd coating thickness, the overall area of Cu-exposed regions decreases, and their distribution gradually shifts toward the FAB bottom [36].

To quantitatively evaluate the Pd-deficient regions on the FAB surface, the exposed Cu area fraction k is introduced as follows:

(3) $$k=S_{1}/S_{2}$$

where *S*_1_ is the area of Cu-exposed regions on the FAB surface, and S_2_ is the total surface area of the FAB. The calculation of the k value in this work is based on the projected area in the direction of observation. Since the FAB is a three-dimensional sphere, the exposed Cu fraction derived from Equation (3) represents a two-dimensional projected area fraction rather than an absolute 3D surface coverage. Image segmentation was performed using a thresholding method and manually verified to ensure consistency.

Based on measurements from Figure 9, the values of k for wires A, B, C, and D are 13 ± 0.8%, 10 ± 0.5%, 9 ± 0.7%, and 6 ± 0.6%, respectively, indicating that increasing Pd coating thickness significantly improves surface coverage uniformity.

**Figure 10:**
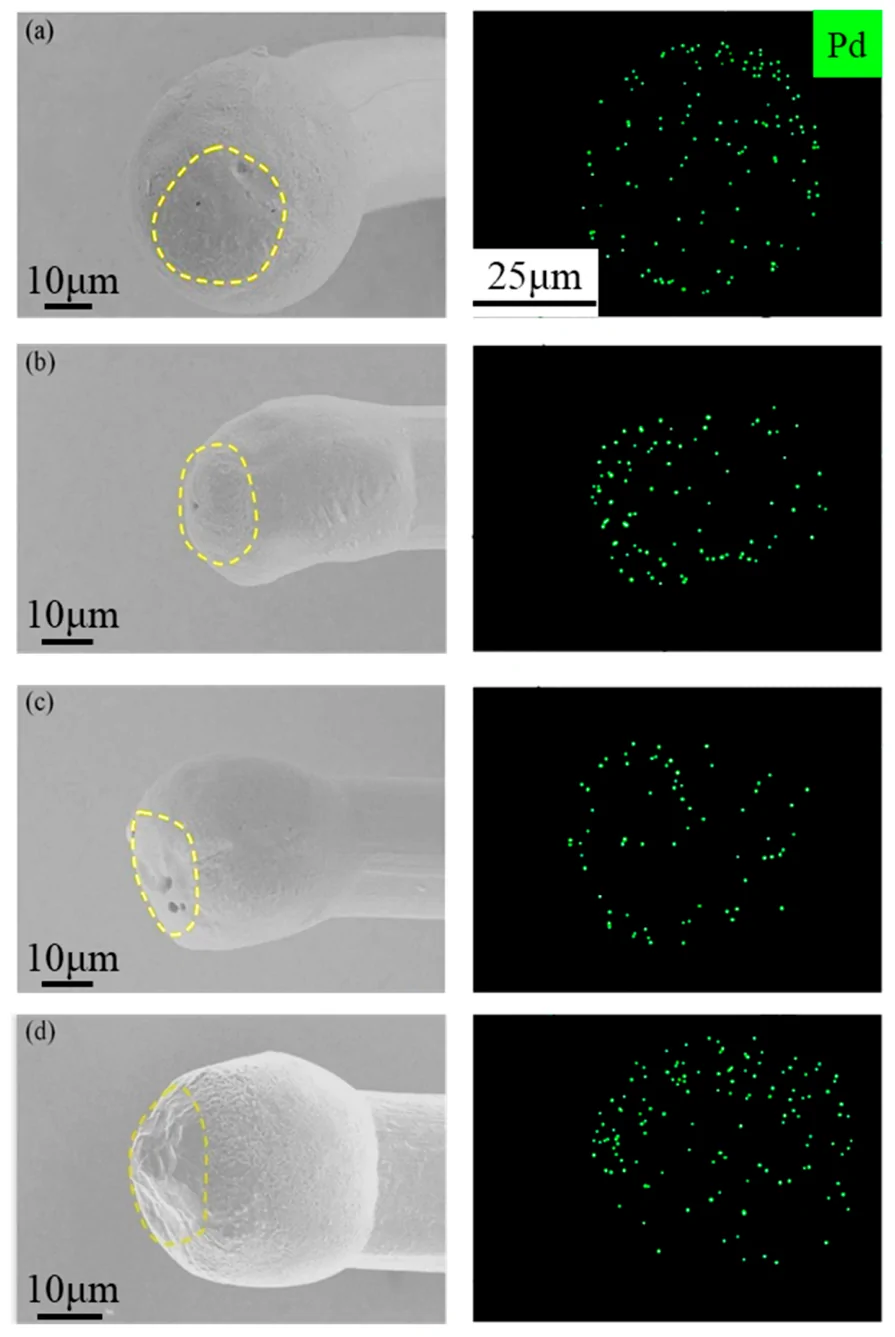
Scanning electron microscopy images of free air balls formed from four wire types after corrosion treatment under a burning current of 44 mA and a burning time of 580 μs, together with the corresponding Pd element distribution on the free air ball surface: (**a**) bonding wire A; (**b**) bonding wire B; (**c**) bonding wire C; (**d**) bonding wire D.

As shown in Figure 10, under a lower burning ball current of 44 mA and a burning ball time of 580 μs, the insufficient melting of the FAB is observed for all samples, resulting in an ellipsoidal morphology. In this condition, Cu-exposed regions are predominantly located at the bottom of the FAB. Meanwhile, the Pd layer is not sufficiently melted or redistributed, and the Pd-deficient cross-section formed after second bond separation is not effectively covered. These results suggest that insufficient energy input restricts Pd redistribution and surface reconstruction, which is a key factor leading to the formation of Cu-exposed regions.

**Figure 11:**
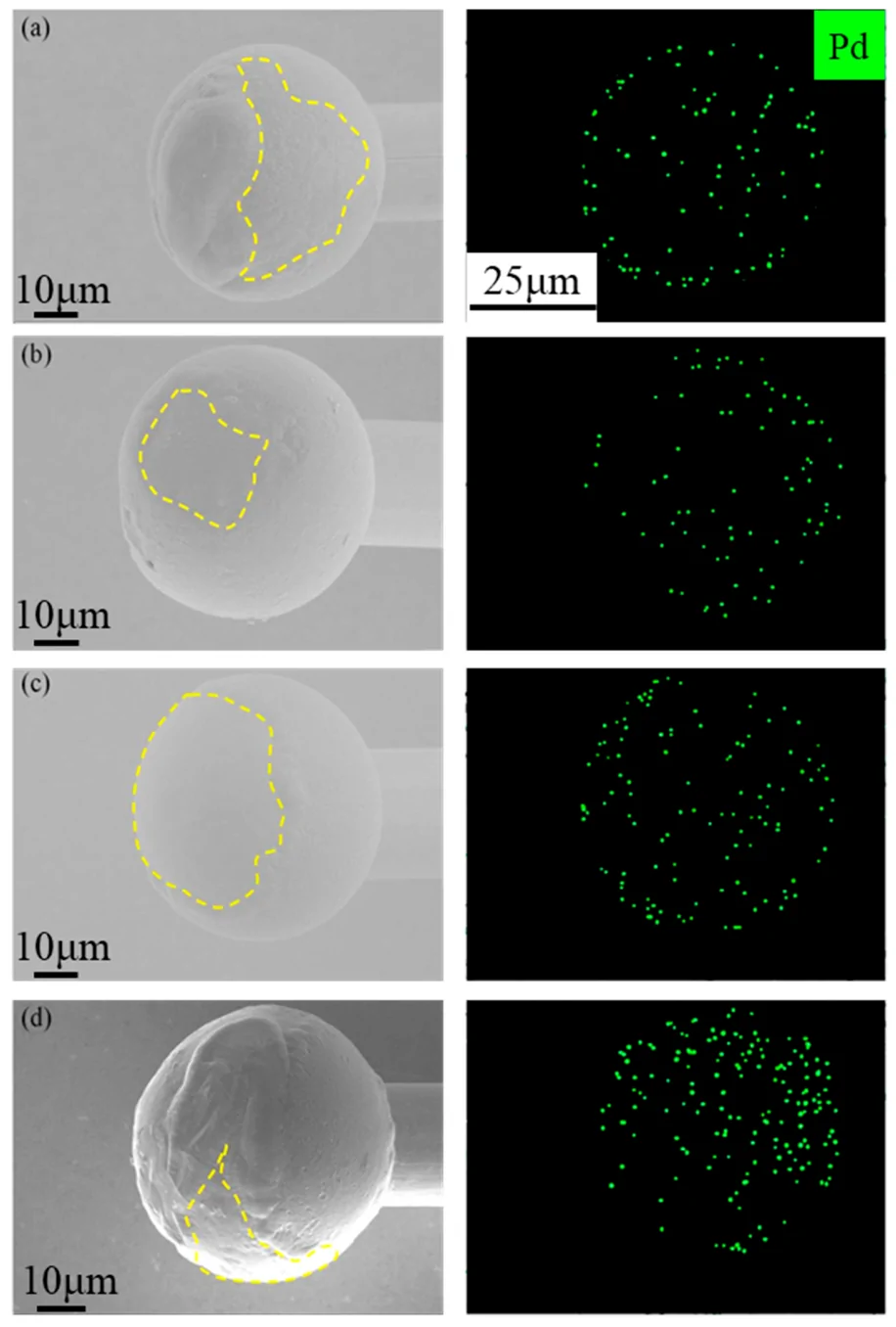
Scanning electron microscopy images of free air balls formed from four wire types after corrosion treatment under a burning current of 64 mA and a burning time of 580 μs, together with the corresponding Pd element distribution on the free air ball surface: (**a**) bonding wire A; (**b**) bonding wire B; (**c**) bonding wire C; (**d**) bonding wire D.

When the burning ball current is increased to 64 mA while keeping the burning ball time at 580 μs, the corrosion morphologies of FABs are as shown in Figure 11. The results indicate that for wires A and C, Cu-exposed regions tend to be more symmetrically distributed, whereas for wires B and D, the distributions become more irregular. Specifically, for wire A, Cu-exposed regions are mainly located in the central and neck regions; for wire B, they are concentrated in the lower-middle region; and for wire C, they further shift toward the bottom of the FAB while exhibiting better axial symmetry.

With increasing burning ball current, the distribution of Cu-exposed regions on the FAB surface exhibits a transition from the bottom region toward the middle and then re-distributes toward the lower-middle and bottom regions. Meanwhile, increasing Pd layer thickness continuously reduces the exposed Cu area and promotes its localization toward the FAB bottom. At 64 mA, higher thermal input leads to the complete melting of both the Cu core and Pd coating, resulting in a strong temperature gradient and intensified molten flow within the FAB. During the EFO process, Pd redistribution is affected by multiple factors, including transient thermal gradients, liquid-phase mixing, and the initial Pd coating distribution. Considering the extremely short duration of the EFO process, liquid-phase convection is expected to contribute more significantly than long-range solid-state diffusion to the observed redistribution behavior.

### 3.3. Internal Pore Structure of FAB and Pd Element Redistribution Behavior

**Figure 12:**
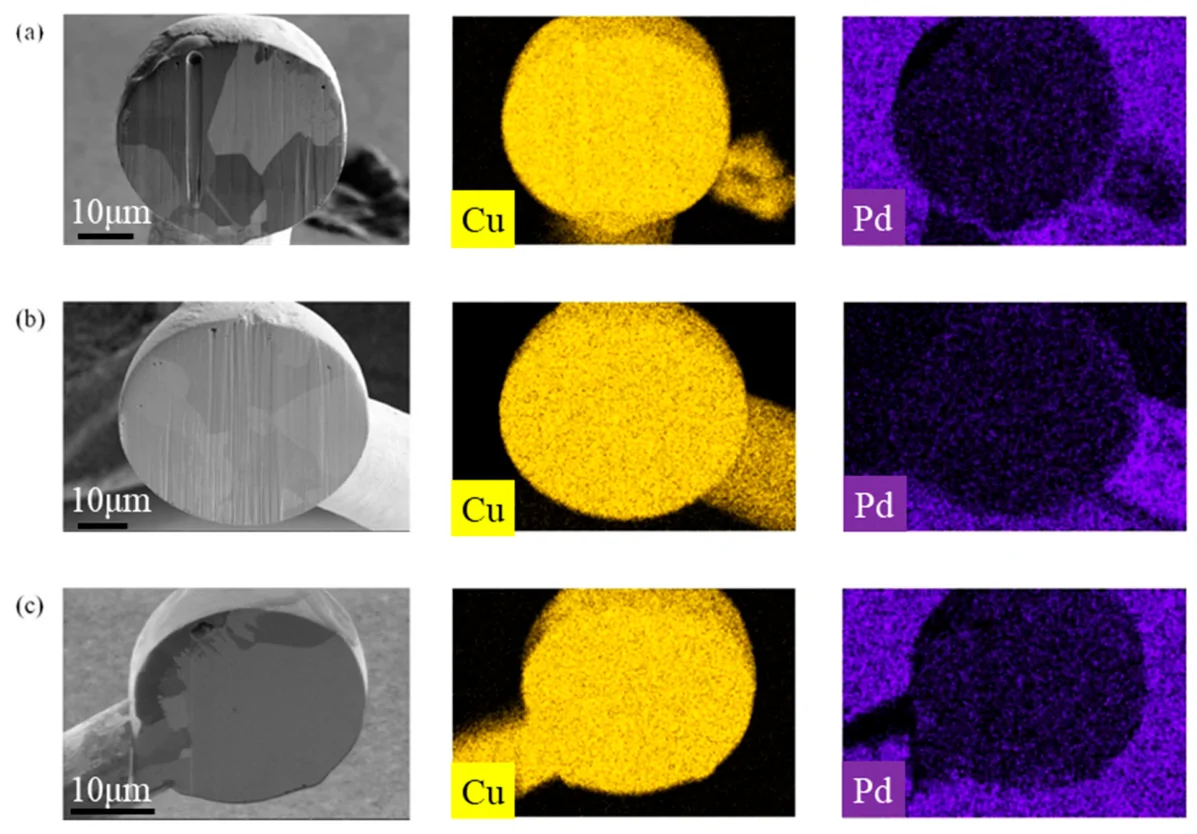
Scanning electron microscopy images of the cross-sectional region of the free air ball formed by bonding wire A under different burning currents at a burning time of 580 μs, together with elemental distribution maps of Cu and Pd in the cross-section: (**a**) 44 mA; (**b**) 54 mA; (**c**) 64 mA.

**Figure 13:**
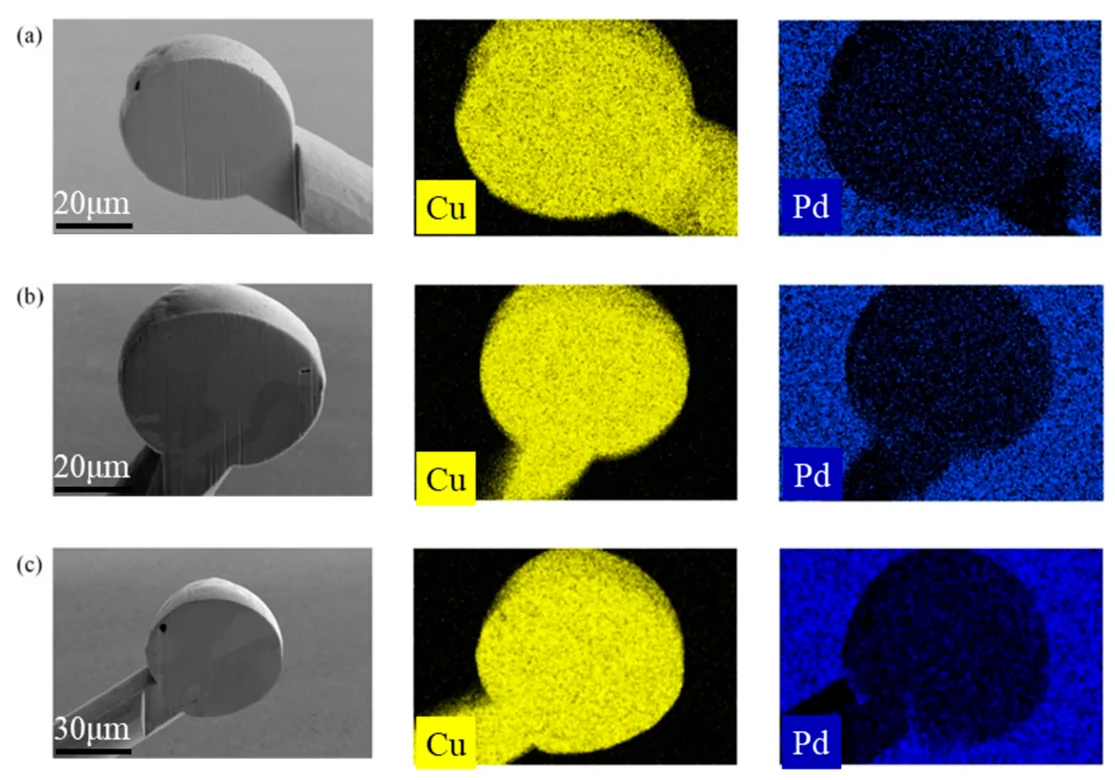
Scanning electron microscopy images of the cross-sectional region of the free air ball formed by bonding wire D under different burning currents at a burning time of 580 μs, together with elemental distribution maps of Cu and Pd in the cross-section: (**a**) 44 mA; (**b**) 54 mA; (**c**) 64 mA.

**Figure 14:**
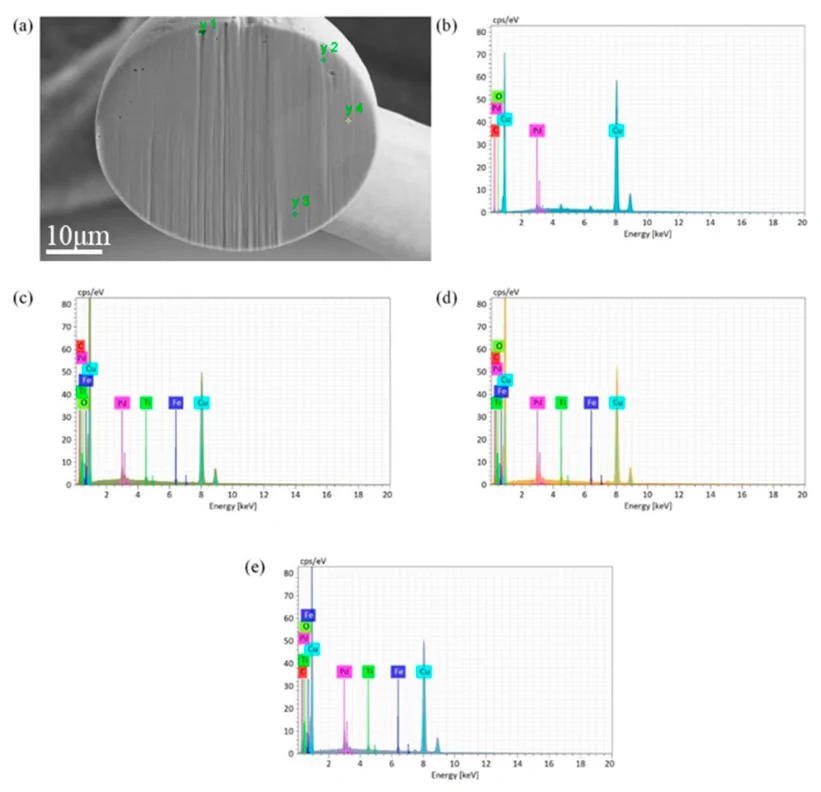
(**a**) Scanning electron microscopy image of the cross-section of the free air ball formed from bonding wire A after cutting; (**b**) the EDS spectrum at position y1; (**c**) the EDS spectrum at position y2; (**d**) the EDS spectrum at position y3; (**e**) the EDS spectrum at position y4.

**Figure 15:**
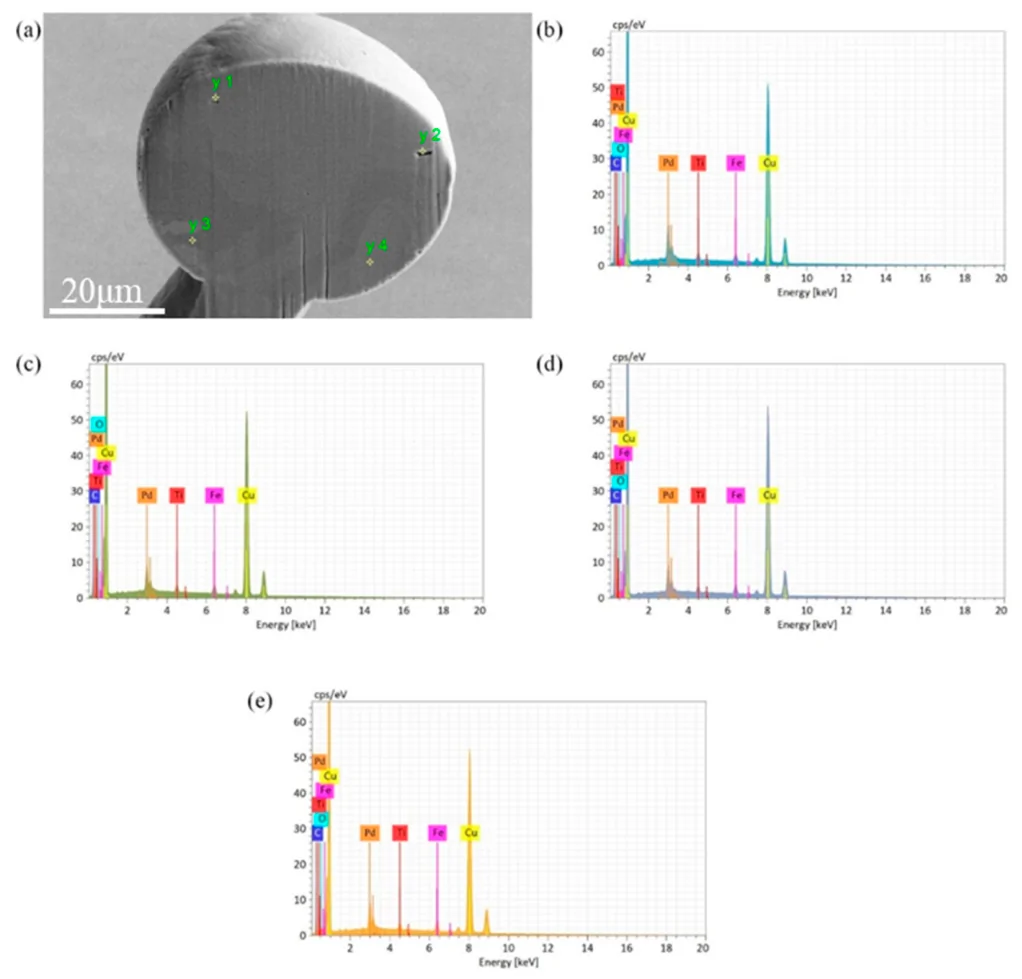
(**a**) Scanning electron microscopy image of cross-section of free air ball formed from bonding wire D after cutting; (**b**) EDS spectrum at position y1; (**c**) EDS spectrum at position y2; (**d**) EDS spectrum at position y3; (**e**) EDS spectrum at position y4.

The EDS point analysis and cross-sectional images presented in Figure 12, Figure 13, Figure 14 and Figure 15 and Table 3, Table 4, Table 5 and Table 6 are selected representative cross-sections. As shown in Figure 12, under a burning current of 44 mA, relatively large pores are mainly located at the bottom region of the free air ball, while a high density of nanoscale pores is distributed in the central region. When the current increases to 54 mA, the large pores are observed to be more centrally located. At 64 mA, they are observed to be further distributed toward the upper region of the free air ball, while dense nanoscale pores remain distributed on both sides of the cross-section. In addition, Pd is relatively uniformly distributed within the free air ball at 44 mA, remains nearly homogeneous at 54 mA, and becomes strongly asymmetric at 64 mA, where Pd enrichment appears on one side of the neck region, whereas no obvious aggregation is observed on the opposite side.

A similar evolution trend is observed for bonding wire D, as shown in Figure 13. At 44 mA, large pores are concentrated at the bottom; at 54 mA, they shift toward the central region; and at 64 mA, they further move to the upper-middle region of the free air ball.

To further clarify the elemental distribution characteristics, EDS analysis was performed on representative regions of the free air ball cross-section of bonding wire A under 580 μs and 54 mA, as shown in Figure 14 and Table 3. The detected C, O, Ti and Fe signals may originate from FIB preparation, sample mounting, or interaction volume effects. Therefore, these elements are not considered in the discussion of Pd redistribution, and only relative Cu/Pd variation is analyzed. The results show that the Cu content at position y1 is significantly higher than at y2–y4, while Pd exhibits the opposite trend. This indicates that large pores are preferentially associated with Cu-rich interfaces, whereas Pd tends to shift along convection channels toward the neck region and nanoscale pore zones.

Under increasing burning current, the compositional variation at position y1 becomes more pronounced (Table 4). When the current increases from 44 mA to 54 mA, Cu content increases significantly, while Pd decreases. When the current further increases to 64 mA, Pd partially recovers while Cu stabilizes, suggesting the secondary redistribution of Pd under strong convective flow. In the y2–y4 regions, Cu first increases and then stabilizes, Pd decreases and then recovers, and C gradually stabilizes.

A similar but weaker trend is observed for bonding wire D, as shown in Figure 15 and Table 5, indicating that a thicker Pd coating suppresses long-range elemental transport through convective flow channels. Compared with bonding wire D, bonding wire A exhibits higher Cu content and lower Pd content at position y1, suggesting that the thicker Pd coating inhibits the segregation of Cu and the enrichment in Pd toward the pore-free region at the FAB bottom. It should be noted that the C signals in the EDS analysis are considered unreliable due to potential contamination during FIB preparation and sample transfer; therefore, C elemental redistribution is not used to infer structural integrity in this study.

As can be seen from Table 6, at 580 μs, increasing current from 44 mA to 54 mA only slightly increases Cu content at y1 in wire D, while Pd remains relatively stable, indicating that a thicker Pd layer suppresses rapid elemental transport under moderate energy input. When the current increases to 64 mA, Cu at y1 rises sharply to 91.42%, while Pd decreases to 0.65%, reflecting rapid elemental redistribution away from the pore-rich region. In contrast, y2–y4 exhibit a consistent trend with the partial recovery of Pd, indicating stronger local flow modulation in these regions.

## 4. Conclusions

This study systematically investigates the influence of electronic flame-off (EFO) burning parameters on free air ball (FAB) formation and the redistribution behavior of Pd on both the surface and interior of FABs, using 1 mil palladium-coated copper wires with four different Pd coating thicknesses. Based on SEM, FIB, and EDS analyses, the following conclusions are drawn:

(1) Under a burning current of 54 mA and a burning time of 580 μs, all samples exhibit preferred FAB morphology, with a diameter-to-wire ratio close to 2, together with improved sphericity and symmetry. This parameter window provides a balanced condition between dimensional stability and morphological integrity and can be considered a common preferred EFO process window based on morphological assessment for wires with different Pd coating thicknesses.
(2) Copper-exposed regions are inevitably present on all FAB surfaces, indicating that the Pd coating cannot fully cover the entire surface after FAB formation. As the Pd coating thickness increases from 60 nm to 120 nm, the exposed Cu area decreases from 13% to 6% and gradually shifts from the upper/middle region toward the bottom of the FAB. This trend demonstrates that increasing coating thickness improves surface coverage uniformity and may provide potential advantages for subsequent bonding reliability evaluation.
(3) The FIB cross-sectional results reveal that internal pore structures undergo significant redistribution under EFO energy input. As the burning current increases from 44 mA to 64 mA, large pores exhibit a distribution shift from the bottom region toward the upper-middle region of the FAB. Meanwhile, obvious Pd enrichment appears in the neck region.
(4) EDS analysis further indicates that large pores are associated with Cu-rich regions, while Pd preferentially shifts toward nanoscale pores and the neck region, forming localized enrichment. With increasing burning current, Cu and Pd elements redistribute more intensively under enhanced melt flow. This demonstrates that higher energy input enhances elemental redistribution intensity within the FAB. In addition, Pd coating thickness significantly affects diffusion behavior and local enrichment characteristics.

## Figures and Tables

**Table 1:** Test scheme of ball burning parameters.

Number	Burning Ball Time (μs)	Burning Ball Current (mA)	Number of Experiments
1	500	44	40
2	540	44	
3	580	44	
4	620	44	
5	660	44	
6	500	49	
7	540	49	
8	580	49	
9	620	49	
10	660	49	
11	500	54	
12	540	54	
13	580	54	
14	620	54	
15	660	54	
16	500	59	
17	540	59	
18	580	59	
19	620	59	
20	660	59	
21	500	64	
22	540	64	
23	580	64	
24	620	64	
25	660	64	

**Table 2:** Free air ball (FAB) diameter, symmetry, roundness, and off-ball angle of different bonding wires under burning ball time of 580 μs and burning ball current of 54 mA.

Parameters	Bond Wire A	Bond Wire B	Bond Wire C	Bond Wire D
$D_{A}$/μm	49.86 ± 0.42	52.82 ± 0.5	49.78 ± 0.38	51.04 ± 0.32
FAB diameter/wire diameter	1.98	2.09	1.98	2.02
Symmetry $D_{R}$	0.99	0.99	1.03	0.99
Roundness $D_{RD}$	0.0088	0.0075	0.0256	0.0086
Deviation angle/°	3°	4°	2°	3°

**Table 3:** Elemental composition (wt.%) at positions y1, y2, y3, and y4 in the cross-sectional cut of the free air ball formed by bonding wire A under a burning time of 580 μs and a burning current of 54 mA.

Positions	Elemental Composition (wt.%)					
	Cu	Pd	C	O	Ti	Fe
y1	85.88	3.24	7.83	0.77	1.11	1.17
y2	77.83	5.04	12.99	1.56	1.24	1.33
y3	75.67	6.93	12.93	1.85	1.19	1.43
y4	76.35	6.51	12.91	1.65	1.26	1.32

**Table 4:** Elemental composition (wt.%) at positions y1, y2, y3, and y4 in the cross-sectional cut of the free air ball formed by bonding wire A under a burning time of 580 μs and a burning current of 44 mA and 64 mA.

Positions	Elemental Composition (wt.%)					
	44 mA			64 mA		
	Cu	Pd	C	Cu	Pd	C
y1	67.48	11.57	15.32	83.10	4.95	6.08
y2	73.18	7.52	13.98	76.61	5.60	13.02
y3	71.85	8.54	14.42	74.07	7.48	13.52
y4	72.80	8.28	13.97	75.19	5.55	13.34

**Table 5:** Elemental composition (wt.%) at positions y1, y2, y3, and y4 in the cross-sectional cut of the free air ball formed by bonding wire D under a burning time of 580 μs and a burning current of 54 mA.

Positions	Elemental Composition (wt.%)					
	Cu	Pd	C	O	Ti	Fe
y1	73.63	8.84	12.88	1.84	1.19	1.61
y2	77.57	6.63	11.36	1.51	1.12	1.81
y3	76.28	7.38	11.77	1.37	1.14	2.06
y4	77.29	6.19	11.92	1.42	1.08	2.01

**Table 6:** Elemental composition (wt.%) at positions y1, y2, y3, and y4 in the cross-sectional cut of the free air ball formed by bonding wire D under a burning time of 580 μs and a burning current of 44 mA and 64 mA.

Positions	Elemental Composition (wt.%)					
	44 mA			64 mA		
	Cu	Pd	C	Cu	Pd	C
y1	70.57	8.91	14.50	91.42	0.65	3.84
y2	77.92	7.03	10.78	72.79	9.76	12.16
y3	76.03	7.97	10.80	73.18	8.66	13.23
y4	77.60	7.75	10.39	69.57	12.37	12.82

## Data Availability

The original contributions presented in this study are included in the article. Further inquiries can be directed to the corresponding author.
